# Modulation of experimental autoimmune encephalomyelitis by endogenous Annexin A1

**DOI:** 10.1186/1742-2094-6-33

**Published:** 2009-11-13

**Authors:** Nikolaos Paschalidis, Asif J Iqbal, Francesco Maione, Elisabeth G Wood, Mauro Perretti, Rod J Flower, Fulvio D'Acquisto

**Affiliations:** 1 William Harvey Research Institute, Barts and the London School of Medicine and Dentistry, Queen Mary University of London, Charterhouse Square, London, EC1M 6BQ, UK

## Abstract

**Background:**

Autoimmune diseases, like multiple sclerosis, are triggered by uncontrolled activation of cells of the immune system against self-antigen present, for instance, in the central nervous system. We have reported novel biological functions for Annexin A1, an effector of endogenous anti-inflammation, to produce positive actions on the adaptive immune system by reducing the threshold of T cell activation. In this study, we investigated the potential modulatory role of Annexin A1 in the development of experimental autoimmune encephalomyelitis, a model of multiple sclerosis.

**Methods:**

Male control C57/BL6 and AnxA1 null mice were immunized subcutaneously with an emulsion consisting of 300 μg of MOG_35-55 _in PBS combined with an equal volume of CFA. Lymph node cells obtained from mice immunized with MOG_33-55 _for 14 days were re-stimulated *in vitro *with MOG_33-55 _(100 μg/ml) for 4 days and the Th1/Th17 cytokine profile measured by ELISA. Spinal cords were processed either to isolate the infiltrated T cells or fixed and stained with haematoxylin and eosin. Statistical analyses were performed using two-tailed, unpaired Student's t tests or ANOVA.

**Results:**

Our results show a direct correlation between Annexin A1 expression and severity of EAE. Analysis of MOG_35-55_-induced EAE development in Annexin A1 null mice showed decreased signs of the disease compared to wild type mice. This defect was significant at the peak of the disease and accompanied by reduced infiltration of T cells in the spinal cord. Finally, analysis of the T cell recall response *in vitro *following stimulation with MOG_35-55 _showed a decrease proliferation of Annexin A1 null T cells, with a significantly reduced Th1/Th17 phenotype, compared to wild type cells.

**Conclusion:**

Together these findings suggest that Annexin A1 null mice have an impaired capacity to develop EAE. Furthermore strategies aiming at reducing Annexin A1 functions or expression in T cells might represent a novel therapeutic approach for multiple sclerosis.

## Background

Multiple sclerosis (MS) is chronic disabling disease caused by malfunction of the immune system. Like many other autoimmune diseases, it is initiated by an uncontrolled T cell response to autoantigens presented in the context of MHC molecules of antigen presenting cells. Several factors have been described as involved in the pathogenesis of MS including environmental, genetic and viral [1]. However, one feature is common to all these cases: the hyperesponsivity of T cells. In MS it is thought that myelin peptides presented by glial cells in the central nervous system (CNS) induce proliferation and activation of Th effector cells. These cells are in turn responsible for the development of the inflammatory reaction and consequent demyelination [2].

Recent views on differentiation of naïve CD4+ T cells in effector Th cells have shown that there are at least 3 different categories (Th1, Th2 and Th17) of effectors cell, a classification mainly based on the type of infection or immune reaction and the cytokine signature produced. Classically, Th1 cells are involved in the cellular-mediated immune reaction and their differentiation is induced upon infection by intracellular bacteria. On the other hand Th2 cells develop during infections with extracellular bacteria and they play a major role in humoral-mediated immune response [3]. Th17 are emerging as the major pathogenic cell lineage responsible for the development of autoimmune and inflammatory disorders [4,5].

Annexin A1 (AnxA1), previously known as lipocortin-1, was originally identified as a phospholipase A2 (PLA2)-inhibitory protein and second messenger of glucocorticoid pharmacological effects [6,7]. Subsequent studies have shown that this protein is also an effector of endogenous inflammatory resolution, where it acts to downregulate neutrophil trafficking and activation, promoting the removal of apoptotic cells by tissue macrophages [8]. However, we have recently demonstrated a novel function for AnxA1 on T cell activation and differentiation [8-10]. Addition of human recombinant (hr)AnxA1 to T cells stimulated with anti-CD3/CD28 increases their activation and favours differentiation into Th1 [11]; conversely, AnxA1^-/- ^T cells display a decreased response to TCR stimulation associated with a marked Th2 phenotype [12]. Analysis of AnxA1 expression in T cells from patients suffering from rheumatoid arthritis showed higher levels of this protein compared to healthy control volunteers [11,13], providing clinical relevance to the role that AnxA1 might play in autoimmune diseases. Together these findings suggest that AnxA1 acts as a positive modulator of T cells and might facilitate the development of autoimmune diseases contributing to aberrant T cell activation.

On these bases, we have investigated here the development of EAE in AnxA1 null mice monitoring macroscopic signs of disease in a temporal fashion, together with histological analysis of spinal cord and *ex-vivo *T cell reactivity upon restimulation with the specific antigen. The results obtained corroborate the hypothesis that blocking AnxA1 function or expression during autoimmune diseases might open new avenues for the therapeutic control of these pathologies.

## Methods

### Reagents

The Myelin Oligodendrocyte Glycoprotein peptide (MOG)_33-55 _(MEVGWYRSPFSRVVHLYRNGK) was synthesized and purified by Cambridge Research Biochemicals (Billingham, UK). Complete Freund's adjuvant containing *Mycobacterium tuberculosis *H37a was purchased from Difco while *Bordetella pertussis *toxin was from Sigma-Aldrich Co (Poole, UK). Unless otherwise specified, all the other reagents were from Sigma-Aldrich Co.

### Mice

Male AnxA1 null mice were previously described [14,15] (9-11 week old) and were backcrossed on a C57BL/6 background for >10 generations and bred at B&K animal care facilities (Hull, UK). Age and gender-matched control C57BL/6 mice were used as control for all experiments. Animals were kept under standard conditions and maintained in a 12 h/12 h light/dark cycle at 22 ± 1°C in accordance with United Kingdom Home Office regulations (Animal Act 1986) and of the European Union directives.

### Induction of EAE

Mice were immunized subcutaneously on day 0 with 300 μl of emulsion consisting of 300 μg of MOG_35-55 _in PBS combined with an equal volume of CFA containing 300 μg heat-killed *M. tuberculosis *H37Ra. The emulsion was injected in both flanks and followed by an intraperitoneal injection of *B. pertussis *toxin (500 ng/100 μl) in 100 μl of saline on days 0 and 2. Mice were observed daily for signs of EAE and weight loss. Diseases severity was scored on a 6-point scale: 0 = no disease; 1 = partial flaccid tail; 2 = complete flaccid tail; 3 = hind limb hypotonia; 4 = partial hind limb paralysis; 5 = complete hind limb paralysis; 6 = moribund or dead animal.

#### Cell proliferation assay

Lymph node cells (10^5 ^cells/200 μl) obtained from mice immunized with MOG_33-55 _for 14 days were stimulated with MOG_33-55 _(50-100 μg/200 μl) for 48 h in 96 well plates. During the last 12 h, cultures were pulsed with 1 μCi of [^3^H]-thymidine (Amersham Pharmacia Biotech, Buckinghamshire, UK) and incorporated radioactivity was measured by automated scintillation counter (Packard Instrument Company, Inc., Illinois, US).

#### Cytokine ELISA

Lymph node cells (10^6 ^cells/ml) obtained from mice immunized with MOG_33-55 _for 14 days were stimulated with MOG_33-55 _(100 μg/ml) for 4 days. Cell supernatants were collected and analyzed for IFN-γ, IL-2, IL-17A and TNF-α content using ELISA kits (eBioscience, Dorset, UK) according to manufacturer's instructions.

#### Isolation of inflammatory cell from the spinal cord

Mice were killed using CO_2_. The spinal cords were expelled from the spinal column with PBS by hydrostatic pressure using a syringe attached to a 21-gauge needle. Tissues were cut in small pieces and passed through cell strainer (70 nm; BD Falcon) using the plunger of a sterile 1 ml syringe. The single cell suspension was centrifuged for 10 min at 390 × g, resuspended in 20 ml of PBS containing 30% of Percoll (Sigma) and overlayed onto 10 ml of PBS containing 70% Percoll. After centrifugation at 390 × g for 20 min, the mononuclear cells were removed from the interphase, washed, and resuspended in FACS buffer (PBS containing 1% FCS and 0.02% NaN_2_) for further analysis.

#### Flow cytometry

Cell samples from Percoll-purified spinal cord tissues or Ficoll-purified lymph nodes were resuspended in FACS buffer containing CD16/CD32 FcγIIR blocking antibody (clone 93; eBioscience) for 30 min at 4°C. Thereafter, cell suspensions were labelled with the FITC-conjugated anti-CD3 (1:100; clone 145 2C11) or anti-F4/80 (1:100; clone BMT) while lymph node cells were stained with anti-CD4-FITC (1:500; clone L3T4) and anti-CD8 (1:1000; clone Ly-2) for 30 min at 4°C, prior to analysis by FACS calibur using CellQuest software (Becton Dickinson). At least 10^4 ^cells were analyzed per sample, and determination of positive and negative populations was performed based on the staining attained with irrelevant IgG isotypes.

#### Histology

Spinal cord tissues were dissected and fixed in 4% neutral buffered formalin for 48 hrs and then incubated with decalcifying solution containing EDTA (0.1 mM in PBS) for 14 days prior to paraffin embedding. Histological evaluation was performed on paraffin-embedded sections sampled at various time points depending on disease severity. Spinal cord sections (5 μm) were deparaffinized with xylene and stained with haematoxylin and eosin (H&E) to asses inflammation. The staining for AnxA1 was performed on frozen sections using anti-AnxA1 (dilution 1:500; Zymed, Invitrogen) and anti-rabbit Ig horseradish peroxidase (HRP)-conjugated antibodies (dilution 1:500; Dako). Double staining for AnxA1 and CD3 or F4/80 was carried out as previously described using FITC-conjugated anti-CD3 (1:100; clone 145 2C11) or anti-F4/80 (1:100; clone BMT). Sections were also counterstained with haematoxylin. In all cases, a minimum ≥ 3 sections per animal were evaluated. Phase-contrast digital images were taken using the Image Pro image analysis software package.

### Statistical Analysis

Prism software (GraphPad software) was used to run all the tests. Statistical evaluations of cell frequency, proliferation and cytokine production were performed using two-tailed, unpaired Student's t tests. ANOVA were applied to analyze the EAE clinical grading. A p value of < 0.05 was considered to be statistically significant. P-values lower than 0.05 were considered significant. Data are presented as mean ± S.E.M of *n *samples per group.

## Results

### AnxA1 expression correlates with the severity of EAE

Previous studies on the role of AnxA1 in the development of EAE in Lewis rat demonstrated a correlation between AnxA1 levels in the spinal cord content and extent of infiltrating mononuclear cells in the CNS [16]. We started off by assessing these phenomena in a mouse model of MS induced by immunization with MOG_35-55_. To this aim, we collected spinal cords and brains of wild type mice immunized with MOG_35-55 _peptide at different stages of the diseases i.e. at day 12 (score 0), day 18 (score 2) and day 20 (score 4) and performed immunohystochemistry for AnxA1 side by side with hematoxylin and eosin staining.

**Figure 1:**
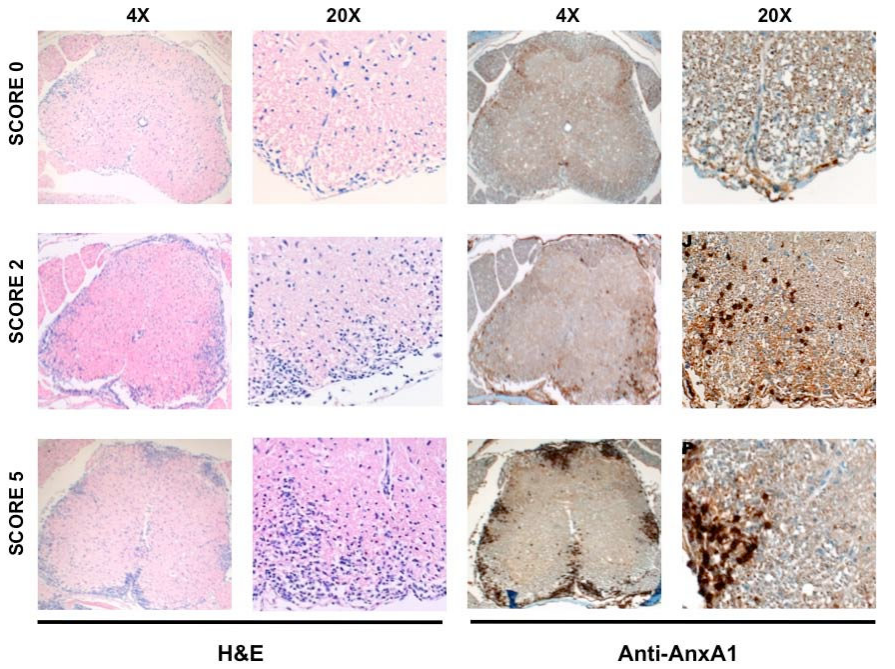
**AnxA1 expression correlates with the severity of EAE**. C57BL/6 mice were immunized with MOG_35-55 _and CFA and spinal cords removed at day 12 (score 0), day 18 (score 2) and (**C**) day 20 (score 4). The sections were stained with hematoxylin and eosin (**A**) or anti-AnxA1 (**B**) as described in Materials and Methods. For each staining, the right panels (20×) show a higher magnification of an area of the left panels (4×). Results representative of 3 experiments.

As shown in Figure 1, spinal cord tissues collected during the induction phase of mice with no signs of disease showed a faint staining for AnxA1 (score 0, Fig. 1A and 1B, respectively). However, with the onset of clinical signs and the appearance of inflammatory infiltrates in the CNS, discrete patches of AnxA1 immunostaining were observed all around the meninges (score 2, Fig. 1A and 1B, respectively). As the disease progressed, an increase in number of AnxA1-positive cellular infiltrate patches was observed (score 4, Fig. 1A and 1B, respectively), suggesting that the infiltration of inflammatory cells expressing high levels of AnxA1 might be correlated with the severity of the disease.

**Figure 2:**
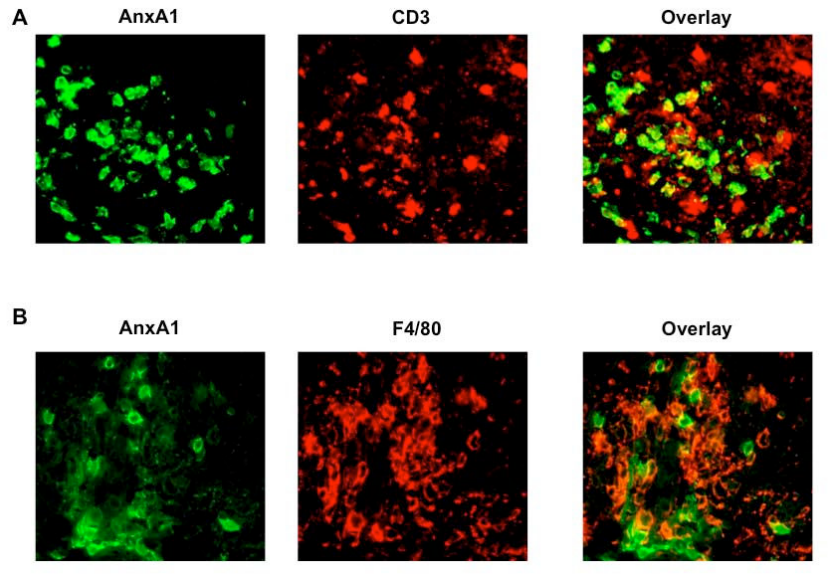
**Cellular phenotype of AnxA1 expressing cells in spinal cord sections of mice with severe EAE**. C57BL/6 mice were immunized with MOG_35-55 _and CFA and spinal cords removed at day 20 (score 4). The sections were stained with anti-AnxA1 and anti-CD3 (**A**) or anti-F4/80 (**B**) as described in Materials and Methods. The right panels show an overlay of the two single stainings on the right. Results representative of 3 experiments.

To identify the cellular sources of AnxA1 immunoreactivity in the spinal cord, we performed double immunofluorescence staining of the sections with anti-AnxA1 and either anti-CD3 (marker for T cells) or anti-F4/80 (marker for macrophages). As expected, we detected a large number of infiltrated T cells and macrophages in the spinal cord sections of mice at the peak of EAE (Fig. 2A and 2B, middle panels, respectively). However, AnxA1 staining in the same sections showed a partial co-localization with both T cells and macrophages without particular preference for one or the other cell types (Fig. 2A and 2B, right panels, respectively).

### AnxA1^-/- ^mice develop an impaired EAE

**Figure 3:**
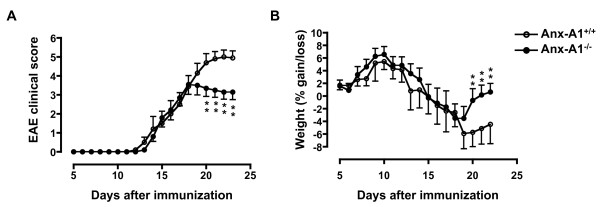
**AnxA1^-/- ^mice developed less severe EAE than AnxA1^+/+^**. C57BL/6 mice were immunized with MOG_35-55 _and CFA and monitored daily for signs and symptoms of EAE (**A**) or weight gain/loss (**B**) for 23 days. Results are means ± SEM (n = 10/group). ** p < 0.01, representative of 3 experiments.

Since AnxA1 expression was upregulated at the peak of EAE, we next investigated the role of this protein on the development of EAE. AnxA1^+/+^and AnxA1^-/- ^mice were immunized s.c. with MOG_35-55 _peptide in CFA on day 0, and then injected i.v. with *B. pertussis *toxin on both day 0 and day 2. Both AnxA1^+/+ ^and AnxA1^-/- ^mice started to develop EAE from day 12 after immunization, reaching peak disease around day 20. However, AnxA1^-/- ^mice had reduced levels of disease compared to AnxA1^+/+ ^(Figure 3A). Interestingly, this was evident and significant only at the later stage of the disease i.e. from day 18 to 23 and onwards.

Studies on animal models of EAE have demonstrated that the acute phase of the disease coincides with weight loss, probably due to anorexia and deficient fluid uptake. Weight measurement of immunized mice correlated with the severity of the clinical score and showed a reduced weight loss - from day 18 onwards - in the AnxA1^-/- ^mice compared to AnxA1^+/+ ^controls (Figure 3B). Further comparison of development of EAE in AnxA1^+/+ ^and AnxA1^-/- ^mice showed a decrease in both the mortality rate and maximum disease score, without differences in the incidence rate or disease onset (Table 1).

**Table 1:** Clinical parameters of MOG_35-55_-induced EAE in AnxA1^+/+ ^and AnxA1^-/- ^mice (mean ± SEM, n = 10/group)

Mice	Incidence^§^	Mortality	Onset day (mean ± SEM)	Max. score (mean ± SEM)
AnxA1^+/+^	100% (10/10)	33.3% (3/10)	16.4 ± 2.3	5.7 ± 0.2
AnxA1^-/-^	100% (10/10)	0% (0/10) **	15.9 ± 1.3	4.3 ± 0.1**

**p < 0.01, representative of 3 experiments

§EAE clinical score equal or greater than 1.

### *In vitro *recall response to MOG_35-55 _in AnxA1^-/- ^mice

**Figure 4:**
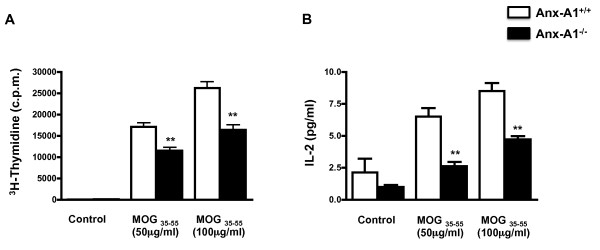
**Analysis of MOG_35-55_-induced T cell proliferative responses in AnxA^/-^**. The graphs shown the incorporation of ^3^H-Thymidine (**A**) and the production of IL-2 (**B**) of lymph node cells obtained from AnxA1^+/+ ^and AnxA1^-/- ^mice immunized with MOG_35-55 _and CFA and sacrificed after 14 days. Cells were stimulated with MOG_35-55 _for 48 hours and pulsed with 1 μCi ^3^H-Thymidine for 12 hours. Cell supernatants were used to measure IL-2 production. Results are means ± SEM (n = 4/group). * p < 0.05, ** p < 0.01, representative of 3 experiments.

T cells play a key role in the development of EAE [17] and AnxA1^-/- ^T cells have an impaired capacity to respond to anti-CD3/CD28 stimulation [12]. In light of these findings, we investigated whether the decreased development of EAE in AnxA1^-/- ^mice was associated with a lower response to antigen-stimulation. Lymph node cells from AnxA1^+/+ ^and AnxA1^-/- ^mice, collected 14 days after immunization, were stimulated *in vitro *with MOG_35-55_. In line with our expectations, AnxA1^-/- ^lymph node cells showed a decreased rate of proliferation and produced lower levels of IL-2 when stimulated with MOG_35-55 _compared to wild-type mice (Figure 4A and 4B, respectively). Similar results were obtained with splenocytes (data not shown).

**Figure 5:**
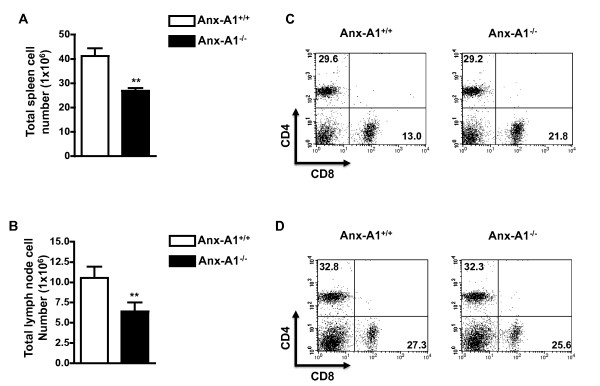
**Spleen and lymph node cellularity of MOG_35-55_-immunized AnxA1^-/- ^mice**. Total cell number of spleen (**A**) and lymph node (**B**) cells obtained from AnxA1^+/+ ^and AnxA1^-/- ^mice immunized with MOG_35-55 _and CFA and sacrificed after 14 days. **C **and **D **show the cytofluorimetric analysis of lymph node cells with anti-CD4 FITC and anti-CD8 PE. Results are means ± SEM (n = 10/group). ** p < 0.01, representative of 3 experiments.

These results on cell proliferation were mirrored in the number of cells recovered from the spleen and the draining lymph nodes of the immunized mice. The total cell count of Ficoll-purified spleen and lymph node mononuclear cells from the same animals, revealed a significant decrease in AnxA1^-/- ^mice compared to controls (Figure 5A and 5B, respectively), with no measurable changes in the percentages of CD4 or CD8 positive cells (Figure 5C and 5D, respectively).

### Reduced MOG_35-55_-specific Th1 and Th17 cytokine responses in AnxA1^-/- ^mice

**Figure 6:**
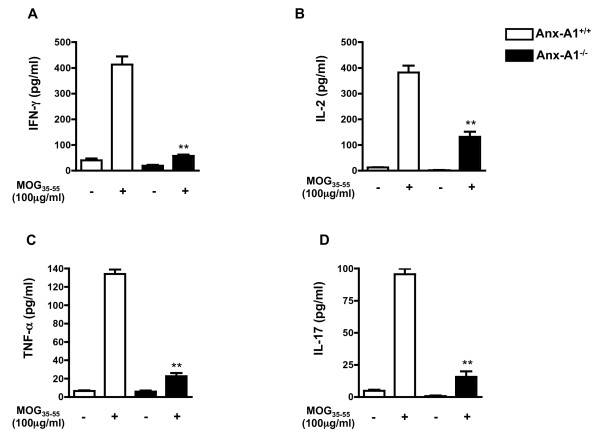
**Impaired Th1 and Th17 cytokine production of MOG_35-55_-immunized AnxA1^-/- ^mice**. Levels of (**A**) IFN-γ, (**B**) IL-2, (**C**) TNF-α and (**D**) IL-17 in the cell supernatants of lymph node cells obtained from AnxA1^+/+ ^and AnxA1^-/- ^mice immunized with MOG_35-55 _and CFA and sacrificed after 14 days. Cells were stimulated with the indicated concentration of MOG_35-55 _for 4 days and the supernatants used for cytokine ELISA. Results are means ± SEM (n = 4/group). * p < 0.05, ** p < 0.01, representative of 3 experiments.

Studies using draining lymph node cells from MOG_35-55 _immunized C57/BL6 mice showed significant changes in Th1 and Th17 cytokine production. Analysis of cytokine production from AnxA1^-/- ^lymph node cells upon re-challenge with MOG_35-55 _for 96 h showed a decreased production of Th1 cytokines IFN-γ, IL-2, and TNF-α compared to wild type cells (Figure 6A-C). Similarly, measurement of Th17 signature product IL-17, revealed decreased levels of this cytokine in AnxA1^-/- ^compared to wild type (Figure 6D).

### T cell infiltration in the nervous system of AnxA1^-/- ^mice during EAE

**Figure 7:**
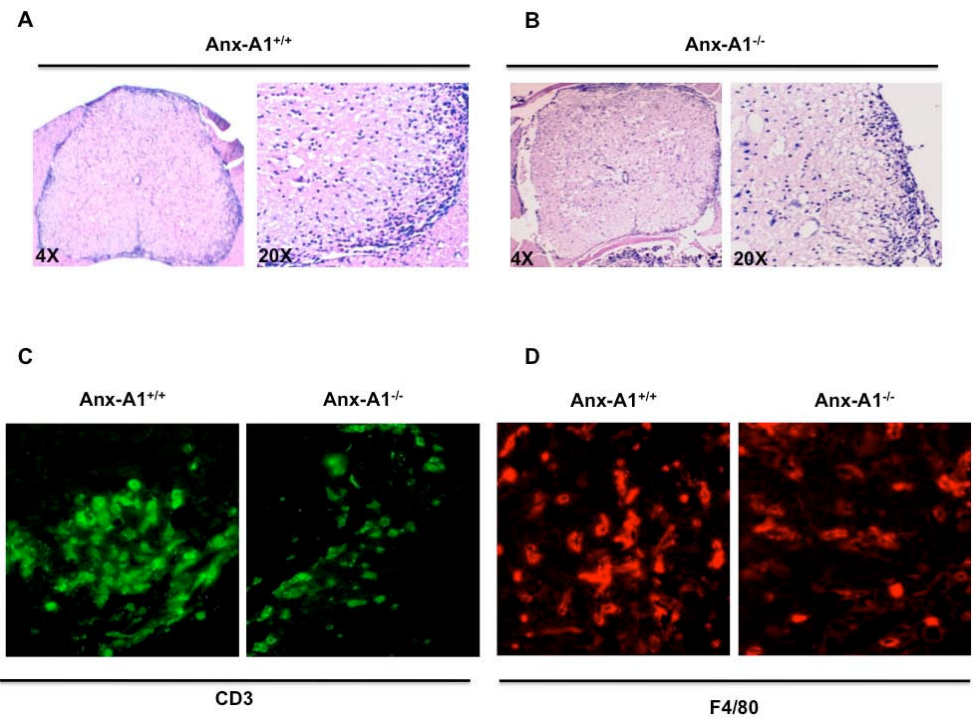
**Histological changes in spinal cord sections of MOG_35-55_-immunized AnxA1^-/- ^mice**. Haematoxylin-eosin staining of spinal cord sections obtained from AnxA1^+/+ ^(**A**) and AnxA1^-/- ^(**B**) mice immunized with MOG_35-55 _and CFA and sacrificed after 22 days. For each staining, the right panels (20×) show a higher magnification of an area of the left panels (4×). Consecutive sections were stained with anti-CD3 (**C**) or anti-F4/80 (**D**) as described in Materials and Methods. Pictures are representative of three separate experiments with similar results.

The reduced signs of EAE in AnxA1^-/- ^mice from day 18 onwards, prompted us to investigate whether there could be a neuro-pathological correlate. The spinal cords of AnxA1^+/+ ^and AnxA1^-/- ^treated mice, collected at day 18 or 22, were analyzed for histological evidence of inflammation. It was found that there were reduced numbers of immune cell infiltrates detected in AnxA1^-/- ^mice compared to AnxA1^+/+ ^animals. (Figure 7A and 7B).

**Figure 8:**
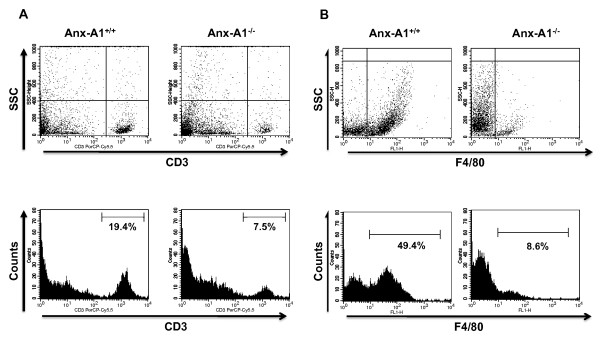
**Cellular phenotype of inflammatory cells present in the spinal cord sections of MOG_35-55_-immunized AnxA1^-/- ^mice**. FACS analysis of CD3 (**A**) and F4/80 (**B**) positive mononuclear cells recovered by Percoll gradient of spinal cord homogenates obtained from AnxA1^+/+ ^and AnxA1^-/- ^mice immunized with MOG_35-55 _and CFA and sacrificed after 14 days. The dot plots and hystograms are from a single mouse and representative of 2 experiments with n = 4 mice. The numbers in the hystograms indicate the percentage of CD3^+ ^and F4/80^+^cells.

The reduced histological signs of inflammation in AnxA1^-/- ^mice were associated with a reduced number of CD3 and F4/80 positive cells infiltrating the CNS (Figure 7C and 7D, respectively). These qualitative analyses were confirmed by FACS measuring the percentages of CD3 and F4/80 positive leucocytes isolated from day 18 spinal cord tissues. Consistent with the immunohistochemistry results, AnxA1^-/- ^mice had about 60 and 80% less T cells and macrophages, respectively, compared to AnxA1^+/+ ^mice (Figure 8A and 8B, respectively).

## Discussion

Emerging evidence over the last five years has shown that AnxA1 exerts a dual function on the innate and adaptive immune systems [8,9]. In the innate immune system, endogenous AnxA1 plays a homeostatic anti-inflammatory role that controls events occurring at the very early stage of the inflammatory process. For instance, studies in AnxA1^-/- ^mice have shown that neutrophils exhibit enhanced transmigration *in vivo *in the inflamed cremaster microcirculation and increased responsiveness *in vitro *upon challenge with PAF, fMLP or PMA [8,9]. Similarly, AnxA1^-/- ^macrophages produce higher levels of TNF-α and IL-6 when challenged with LPS either *in vitro *or *in vivo *[18].

Investigation on the role of AnxA1 in the adaptive immune system provided us with an opposite scenario. AnxA1^-/- ^T cells showed an impaired capacity to proliferate upon anti-CD3/CD28 stimulation and a skewed Th2 phenotype when differentiated *in vitro *[12]. Consistent with this, when we investigated the immune response of AnxA1^-/- ^mice in a model of allergic peritonitis, we observed an increased recruitment of eosinophils in the cavity upon challenge with the ovalbumin [12]. Most interestingly, we also found that AnxA1 plays an unpredicted proinflammatory role in chronic autoimmune diseases. Administration of human recombinant AnxA1 during the immunization phase of the collagen-induced arthritis model exacerbates signs and symptoms of diseases [11].

These results, together with the investigations on the innate immune system, suggest different effects of AnxA1 in inflammatory diseases depending on the relative contribution of the innate and adaptive arms of the immune systems. With this idea in mind, we approached this study knowing that the phenotype of EAE in AnxA1^+/+ ^mice could not readily be predicted.

MOG_35-55_-induced EAE is a model for autoimmune demyelination of the central nervous system and it has been widely used to investigate pathogenic mechanisms responsible for the development of MS. Myelin-reactive T cells are considered an immunological hallmark of both EAE and MS and thought to be the driving force for the recruitment of inflammatory cells in the CNS. These recruited cells include mainly macrophages especially in the C57BL/6 mouse strains.

Our results on the development of EAE in the AnxA1^+/+ ^mice show a decreased capacity to fully develop signs of disease. This was particularly significant at the later stage i.e. when the mice start to show signs of full paralysis. Hystological analysis of the spinal cord supported these results and showed a reduced level of T cell and macrophage infiltration in the AnxA1^-/- ^mice compared to wild type controls.

We hypothesized that the reduction of clinical signs of EAE in AnxA1^-/- ^mice might be due to defect in the activation and expansion of encephalitogenic T cells. The results confirmed our expectation and showed a reduced number of cells in lymph nodes of AnxA1^-/- ^mice as well as a reduced *in vitro *recall proliferative response to MOG_35-55_. In agreement with these data, when we measured the total number of infiltrated T cells in the spinal cord of AnxA1^-/- ^mice, a significant decrease in the number of CD3^+ ^cells was observed. Collectively these data indicate important support properties of endogenous of AnxA1 in modulating T cell activation in this model.

Several explanations can be provided for the AnxA1^-/- ^mice phenotype. Studies from our lab have shown that AnxA1^-/- ^T cells acquire a marked Th2 - but reduced Th1 and Th17 -phenotype when differentiated *in vitro *under optimal Th1, Th2 or Th17 skewing conditions [19]. Here we confirm these results and show that *in vitro *stimulation of lymph node cells from MOG_35-55_immunized mice with the same antigen produced reduced amounts of Th1 (TNF-α, IL-2 and IFNγ) and Th17 (IL-17) cytokines. Analysis of Th2 cytokine IL-4 and IL-5 showed almost double basal production in AnxA1^-/- ^lymph node cells compared to AnxA1^+/+ ^but no further increase upon MOG_35-55 _stimulation (data not shown). This might be due to the fact that fully differentiated Th2 cells appear during the late remission stage of the disease [20,21], while our analyses have been carried out soon after the onset of the disease.

Studies on Th cell differentiation during the development of EAE have shown the involvement of Th1 and Th17 cells during the first phase. However, controversial results present in the literature on this aspect do not provide a conclusive answer on what would be the exact role(s) of Th1 or Th17 [22]. Indeed, administration of IFN-γ exacerbates signs of disease in MS patients [23] and adoptive transfer of Th1 cells effectively induces EAE in mice [24,25]. On the other hand, mice deficient for IL-12 (p35) [26], IFN-γ [27] and TNF-α [28,29] showed no overt impairment in the development of EAE. Similarly, multiple sclerosis lesions contain high levels of IL-17 [30] and, in animal models, adoptive transfer of Th17 cells induce a more severe EAE compared to the lesions produced by the transfer of Th1 cells [31]. This conclusion, again, is in contrast with investigations where overexpression or ablation of IL-17A, specifically in T cells, had no effect on the development of EAE [32].

Our results showing an impaired production of both Th1 and Th17 cytokines in AnxA1^-/- ^deficient T cells suggest that the downstream events elicited by this protein might be shared by both the Th1 and Th17 pathways. However, further studies are needed to verify this hypothesis. One possibility might be that the decreased strength of TCR signalling observed in AnxA1^-/- ^T cells contributes to the inhibition of Th1 and Th17 development [17,33]. Most interestingly, an elegant study by Juedes et al. has shown that in the MOG_35-55_-induced EAE in C57/BL6 mice the early infiltration of Th1 cells is the key to sequential cascade of events i.e. activation of microglia, induction of VCAM and ICAM and finally traffic of mononuclear cells across the endothelium. Our phenotypic characterization of the cellular infiltrates of the MOG_35-55 _immunized AnxA1^-/- ^confirmed these results and showed a reduced number of macrophages compared to wild type mice reinforcing the hypothesis that the reduced development of Th1 cells in the AnxA1^-/- ^mice might be responsible for this effect.

Previous studies on the effects of a truncated version of human recombinant AnxA1 (amino acid 1-188) on the development of EAE in Lewis rat have shown a significant inhibitory effects on mild but not severe EAE [34]. These apparently contrasting results can be explained by the fact that in this study the authors tested the well-known antinflammatory action of exogenously administered AnxA1 by administering the recombinat protein intracerebroventricularly at the onset and throughout the peak of the disease. Interestingly, the same study showed that intracerebroventricular administration of a neutralizing antibody against AnxA1 did not modify the development of EAE [34]. Together these results support our findings showing a prominent role of endogenous AnxA1 in influencing the activation of the immune system that precedes the development of EAE.

Previous studies on role of AnxA1 in the development of EAE demonstrated that the cerebellum and spinal cord content of AnxA1 correlated with appearance of infiltrating lymphocytes and macrophages in the CNS [16,34,35]. We confirmed these results also in this model of MOG_35-55 _induced EAE. Immunohystochemistry for AnxA1 in spinal cord of wild type mice showed a faint staining during the induction phase of the disease. However, with the onset of clinical signs and the appearance of inflammatory infiltrates in the CNS, a marked increase in AnxA1 immunostaining was observed. This was maximal at the peak of the symptoms and suggested that the infiltration of inflammatory cells expressing high levels of AnxA1 might be correlated with the severity of the disease and the consequent tissue damage. In agreement with this hypothesis, previous studies have shown an increase in AnxA1 content in post-mortem CNS tissue samples from MS patients, in particular in the diseased white matter as well as in multiple sclerosis plaque tissue [36].

## Conclusion

In conclusion, this study presents novel evidences of pivotal roles for AnxA1 in a mouse model of multiple sclerosis. Based on the data produced, we propose that AnxA1 deficient T cells might be responsible for the failure to recruit significant number of inflammatory cells into CNS. More analysis is needed to characterize the phenotype of the T cells infiltrating into the CNS in order to further understand the molecular mechanisms by which AnxA1 influences the development of EAE. Nevertheless, our results clearly suggest that the level of expression of this protein in T cells may have a causal function. Future studies on the identification and generation of neutralizing antibodies against AnxA1, currently under development, will provide us the opportunity to validate novel therapeutic approaches for the treatment of multiple sclerosis that target AnxA1 expression or function.

## Competing interests

The authors declare that they have no competing interests.

## Authors' contributions

Design of studies NP, FDA. Experimental induction of EAE, NP and preparation of tissues NP, AJI, FM, EGW, FDA. Writing/reviewing of manuscript NP, MP, RJF, FDA. All authors have read and approved the final manuscript.
